# The Overexpression of NALP3 Inflammasome in Knee Osteoarthritis Is Associated with Synovial Membrane Prolidase and NADPH Oxidase 2

**DOI:** 10.1155/2016/1472567

**Published:** 2016-09-29

**Authors:** Denise Clavijo-Cornejo, Karina Martínez-Flores, Karina Silva-Luna, Gabriela Angélica Martínez-Nava, Javier Fernández-Torres, Yessica Zamudio-Cuevas, Mónica Guadalupe Santamaría-Olmedo, Julio Granados-Montiel, Carlos Pineda, Alberto López-Reyes

**Affiliations:** ^1^Synovioanalysis Molecular Laboratory, Instituto Nacional de Rehabilitación “Luis Guillermo Ibarra Ibarra”, Secretaria de Salud, Calzada Mexico-Xochimilco No. 289, Col. Arenal de Guadalupe, 14389 Tlalpan, Mexico City, Mexico; ^2^Musculoskeletal and Articular Ultrasound Diploma Course, Instituto Nacional de Rehabilitación “Luis Guillermo Ibarra Ibarra”, Secretaria de Salud, Calzada Mexico-Xochimilco No. 289, Col. Arenal de Guadalupe, 14389 Tlalpan, Mexico City, Mexico; ^3^Biological and Health Sciences PhD Program, Universidad Autónoma Metropolitana Iztapalapa, Avenida San Rafael Atlixco 186, Col. Vicentina, 09340 Iztapalapa, Mexico City, Mexico; ^4^Tissue Engineering, Cell Therapy and Regenerative Medicine Research Unit, Instituto Nacional de Rehabilitación “Luis Guillermo Ibarra Ibarra”, Secretaria de Salud, Calzada Mexico-Xochimilco No. 289, Col. Arenal de Guadalupe, 14389 Tlalpan, Mexico City, Mexico; ^5^Instituto Nacional de Rehabilitación “Luis Guillermo Ibarra Ibarra”, Secretaria de Salud, Calzada Mexico-Xochimilco 289, Col. Arenal de Guadalupe, 14389 Tlalpan, Mexico City, Mexico

## Abstract

Osteoarthritis is characterized by the presence of proinflammatory cytokines and reactive oxygen species. We aimed to clarify the role of prooxidant enzyme content at the synovial membrane level and how it correlates with the inflammatory process in patients with knee osteoarthritis (KOA). In synovial membranes from KOA patients and control group, we analyzed the protein content of prooxidant enzymes such as Nox2, xanthine oxidase (XO), and prolidase as well as the proinflammatory NALP3. Results show that protein content of prolidase and Nox2 increased 4.8- and 8.4-fold, respectively, and XO showed an increasing trend, while the NALP3 inflammasome increased 5.4-fold with respect to control group. Levels of prolidase and XO had a positive correlation between the levels of NALP3 and Nox2. By principal component analysis the protein expression pattern by study groups was evaluated. Three clusters were identified; protein expression patterns were higher for clusters two (prolidase) and three (XO and Nox2) between KOA patients and controls. Data suggest that prooxidant enzymes increase in synovial membrane of KOA patients and may contribute to the inflammatory state and degradation of the articular cartilage.

## 1. Introduction

The World Health Organization estimates that 9.6% of men and 18.0% of women aged over 60 years have symptomatic osteoarthritis (OA) [1], defined as a chronic degenerative disease characterized by hyaline articular cartilage breakdown, intra-articular inflammation, and structural joint changes [2]. OA, particularly knee osteoarthritis (KOA), represents a major cause of pain and locomotor disability worldwide [3–6]. Not only does OA involve articular cartilage damage, but also other joint structures participate in the degenerative process, leading to the concept of whole-joint disease [7–9]. The pathophysiological aspects involved in joint damage include the increase of proinflammatory cytokines and reactive oxygen species (ROS) [10]. In normal conditions ROS play a key role in cellular physiology, such as second messenger and gene expression regulation [10–12]; however, ROS overproduction and decrease in antioxidant enzymes lead to oxidative stress [13, 14], which may promote cell damage [11, 15] or extracellular matrix degradation [16, 17] that finally results in chondroptosis and progression of OA [18–20].

The joint has different sources of ROS production [21], such as nicotinamide adenine dinucleotide phosphate oxidase (Nox) [22, 23] and xanthine oxidase (XO) [24]. Recently, the proline metabolism was involved in ROS production [25, 26], in which the prolidase, a rate-limiting enzyme, has a key role. Consequently, ROS overgeneration in the synovial membrane is associated with the inflammatory process mediated by NALP3 inflammasome, implicated in the development of KOA [27, 28].

The aim of this work was to clarify the role of prooxidant enzymes, Nox2, XO, and prolidase, in the inflammatory process that takes place in the synovial membranes of KOA patients.

## 2. Methods

### 2.1. Patients

The study was carried out in 60 subjects undergoing total knee replacement for OA under the care of the inpatient rheumatology and orthopedic surgery departments at the Instituto Nacional de Rehabilitación “Luis Guillermo Ibarra Ibarra”; KOA was diagnosed according to American College of Rheumatology criteria [29]. The Kellgren and Lawrence radiographic scoring system was used to classify KOA [30]. Healthy subjects who underwent elective knee arthroscopy for anterior cruciate ligaments injury were included as a control group. Synovial membrane remnant tissue samples were obtained during the surgical procedure.

The clinical, sociodemographic, and anthropometric data, including body mass index, were obtained from all study subjects. The number of leukocytes in peripheral blood from all participants was obtained from routine laboratory investigations during their preoperative evaluation.

The study fulfilled all criteria contained in the Declaration of Helsinki and was approved by the Ethics and Research Committee of the Instituto Nacional de Rehabilitación “Luis Guillermo Ibarra Ibarra” (Ref. number 42/13). All participants were formally informed about the study and consented in writing to participate.

### 2.2. Western Blot Analysis

Total protein was isolated from synovial membrane tissue samples. Analysis of its protein content was performed by Western blot as previously reported [31, 32]. Prolidase, Nox2, XO, and NALP3 antibodies from Abcam (Ab108980, Ab-5826, Ab109235, and Ab51952, resp.) were used. Loading normalization was performed by anti-actin antibody (Sigma, A3854). The blots were scanned with Amersham Imager 600 RGB (GE) and densitometry analysis was performed with ImageQuant TL 8.1 Software.

### 2.3. Statistical Analysis

Clinical, sociodemographic, and anthropometric data and relative protein concentration were compared between study groups by Mann–Whitney *U* test and Fisher exact test when appropriate.

To evaluate the correlation between relative levels of the proteins of interest Spearman correlation coefficients were determined using Bonferroni correction adjusted significant level. Furthermore, the correlations were assessed stratifying by study group. To expose the degree of relation that Nox2 and prolidase have over NALP3 protein content in the synovial membrane a linear regression analysis was performed.

Principal component analysis (PCA) [33] was performed to assess the possible components of proteins measured and whether they differed between KOA patients and controls. An orthogonal rotation was used, and only the components with eigenvalues >1.0 were taken into account. Variables were categorized per component when their loading scores were >0.5. The variance value of the component was predicted for each subject and compared by study group by Mann–Whitney *U* test.

Statistical analyses were performed with Prism v6.01 (GraphPad Software Inc., California, USA) and STATA v12.1 (Stata Corporation, College Station, TX, USA). All *p* values lower than 0.05 were considered as significant.

## 3. Results

The clinical and demographic data are shown in Table 1. As expected, healthy subjects were younger than KOA patients, with a median of 35 years versus 67 years, respectively (*p* < 0.01). Both KOA patients and control group had similar body mass index (BMI) (median of 25.6 and 27.5, resp., *p* = 0.48). Gender proportion between study groups behaves differently, with mostly males (70%) in the control group and females (79%) in the KOA group (*p* = 0.01). No differences between the groups were found in the number of their peripheral blood leukocytes (*p* = 0.78). None of the study group participants presented leukocytosis.

**Figure 1:**
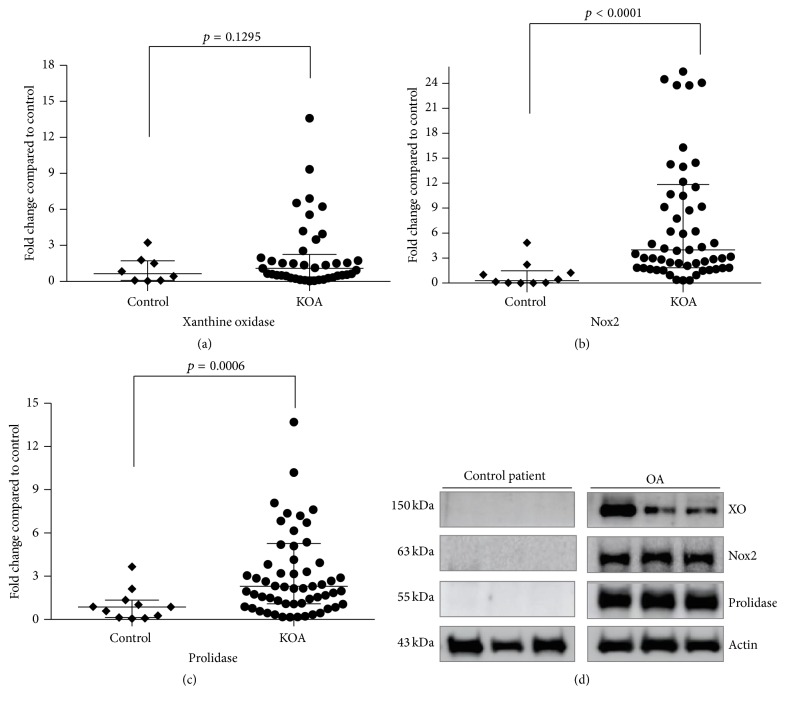
Prooxidants enzymes are overexpressed in KOA patients. The graphics show the densitometry analysis of protein expression of XO (a), Nox2 (b), and prolidase (c) in comparison to the control group. Results are shown as the mean ± SEM (*p* < 0.001 and *p* < 0.0001). Representative Western blot of protein levels relative to actin was used as internal control (d).

**Figure 2:**
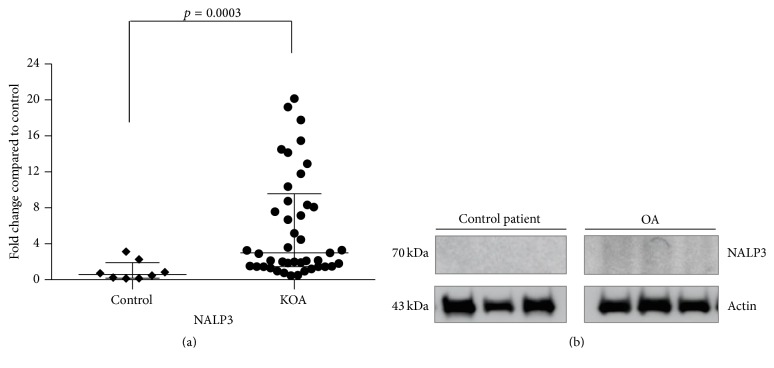
Inflammatory state present in KOA patients. Densitometry analysis of protein expression of NALP3 in comparison to the control group (a). Results are shown as the mean ± SEM (*p* < 0.001). Representative Western blot of protein levels relative to actin used as internal control (b).

To assess the status of prooxidant enzymes in synovial membrane tissue from KOA patients, the protein content of XO, Nox2, and prolidase was analyzed by Western blot (Figure 1(d)). XO displayed only a tendency to have a higher relative content in KOA patients (Figure 1(a)). However, Nox2 (*p* < 0.0001) and prolidase (*p* = 0.0006) showed 8.4- and 4.8-fold change, respectively, when compared to the control group (Figures 1(b) and 1(c), resp.). Additionally, assessment of the NALP3 inflammasome protein (Figure 2(b)) shows a 5.4-fold increased content relative to the control group (*p* = 0.0003) (Figure 2(a)).

We use the correlation coefficient to test whether there is a linear relationship between the prooxidant protein content as a whole and NALP3 inflammasome in KOA patients. A positive correlation between the levels of prolidase and XO (*ρ* = 0.46, *p* = 0.002) and between the levels of NALP3 and Nox2 (*ρ* = 0.50, *p* = 0.001) was found. When these correlations were assessed in the KOA patients group, the correlation between the levels of prolidase and XO was the only one that showed statistical significance *ρ* = 0.50, *p* = 0.02). We could not detect any significant correlation in the control group.

The linear regression analysis allowed us to know how much the NALP3 protein content increases by each unit of NOX2 protein and prolidase. We found that, in the synovial membrane by each Nox2 relative unit, NALP3 increases 0.08 relative units adjusting by age and gender (*β* = 0.08; 95% CI = 0.04–0.11; *p* < 0.001). In the same way, we found that in the synovial membrane by each prolidase relative unit NALP3 increases 0.51 relative units adjusting by age and gender (*β* = 0.51; 95% CI = 0.33–0.69; *p* < 0.001).

**Figure 3:**
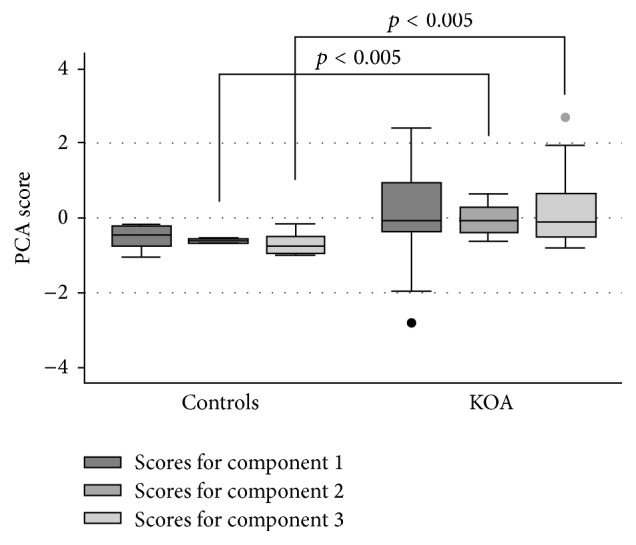
Variability in protein expression in each sample. The analysis was performed by principal component analysis. The graphic shows three components of protein expression, comparing the control group and the patients group. The first component includes NALP3, Nox2 and XO, the second prolidase, and the third XO and Nox2 proteins expression patterns (*p* < 0.005).

By PCA we identified the variables per sample of three components that explained the 82% of the total variance observed overall. Component one was composed of NALP3, Nox2, and XO levels. Component two included only prolidase levels. Component three was composed of XO and Nox2. The scores for the components two and three behaved differently between controls and KOA patients, whose scores were higher for both components than the observed in controls (*p* < 0.005) (Figure 3).

## 4. Discussion

The synovial membrane has important functions in joint homeostasis, including lubrication of cartilage, control of synovial fluid volume, and nutrition of chondrocytes [15]. This specialized tissue experiences important changes in OA, contributing to the whole-joint degeneration process via the production and release of inflammatory effectors [34–36]. Additionally, synovial tissue has been implicated in ROS overproduction [10, 37] and the decrease of antioxidant enzymes leading to oxidative stress [13, 14]. The XO-mediated ROS production triggered by the synovial membrane participates in a series of early injurious events observed in acute knee joint trauma as reported by Stabler et al. [24]. However, our study in patients with advanced KOA showed only a tendency to increase the XO protein expression (Figure 1(a)), suggesting that XO may not be the main source of ROS in KOA.

Currently, it is well known that Nox2, a Nox homologue, plays a key role in bacterial killing and the oxidative burst in phagocytic cells [38, 39]. However, Nox2 is also expressed in nonphagocytic cells, such as endothelial cells, [40], vascular smooth muscle cells [41], and hepatocytes [32, 42] and in chondrocytes from joints of patients with inflammatory arthritis [43, 44]. Recently, the presence of Nox2 was described in the synovial membrane of KOA [45, 46]. However, the overexpression and implications of these enzymes have scarcely been studied in the synovial membrane of KOA. Our data show that Nox2 is increased in KOA patients as compared with the control group (Figure 1(b)), suggesting that Nox2 protein can potentially be involved in the excess ROS production and oxidative stress associated with the condition.

Besides Nox2, study results showed that prolidase overexpression in the synovial membrane from KOA patients (Figure 1(c)) might contribute with ROS overproduction and excess free radical production involved in joint damage. It is well known that prolidase is the only enzyme that specifically cleaves imidodipeptides with carboxyl terminal proline or hydroxyproline at the final stage of collagen degradation in order to recycle the amino acids for collagen resynthesis [25, 26]. As reported by Phang et al., the proline metabolism generates ROS as a regulated manner, but an abnormal production can allow oxidative stress [47, 48]. Therefore, the prolidase enzyme is a rate-limiting enzyme in proline metabolism, which led us to propose that this enzyme plays a role in ROS production in KOA.

It has been reported that, in serum of KOA patients, the ROS are increased and the prolidase activity is decreased, suggesting that the activity of this enzyme is related to the progression of the disease because there is no collagen recycling process [37, 49]. However, the prolidase expression in the synovial tissue remains sparsely studied in KOA. Fitowska et al. reported that, in synovial membrane tissue of patients with hip OA, the prolidase activity is significantly higher compared with the control group [50]. In concordance with this, our study provides evidence that prolidase content in the synovial membrane is increased in KOA patients compared with control group; this data suggests that prolidase-mediated collagen degradation in the joint damage may be involved in the ROS overproduction in KOA disease.

Oxidative stress is known to play a key role in proinflammatory responses, mainly leading to NALP3 inflammasome activation [51–53]. Current evidence suggests that proinflammatory cytokines are responsible for the catabolic process occurring in the pathological tissue. In KOA, the synovia is involved in the production of proinflammatory effectors that are diffused into the cartilage through the synovial fluid, where they activate the chondrocytes to produce more proinflammatory cytokines [7, 34, 54]. Our data show an increase in the protein expression of NALP3 (Figure 2), which may be related to the chronic inflammatory state present in KOA and rheumatoid arthritis patients [28].

Correlation analyses were performed to examine the relationships between prooxidant enzymes and the NALP3 inflammasome in KOA patients. Data show that there is a positive correlation between inflammasome NALP3 and Nox2, which is similar to data previously reported [51, 55, 56] showing that Nox2-mediated ROS production is involved in the NALP3 activation leading to an inflammatory state in the osteoarthritic joint. Even though we did not find a correlation between prolidase and NALP3, data shows that the enzyme is increased in the KOA patients with respect to the control group suggesting its importance. Statistically, the correlation analysis indicates the degree in which two variables are related. In that sense, these results suggest that protein content of Nox2 in the synovial membrane is related to NALP3 protein content. Nevertheless, these correlations coefficients do not show the exact value in which the protein content of NALP3 is being increased by Nox2 protein. To further expose the degree of relation that Nox2 and prolidase have over NALP3 protein content in the synovial membrane we performed a linear regression analysis. This analysis allowed us to know how much the NALP3 protein content increases by each unit of NOX2 protein and prolidase. By each Nox2 relative unit, NALP3 increases 0.08 relative units; in the same way prolidase increases 0.51 relative units adjusting by age and gender. These data suggest the relation between the prooxidant enzymes and the inflammation mediated by NALP3.

Until now, we have shown that increases in Nox2, prolidase, and NALP3 are involved in KOA; nevertheless, we were interested in explaining the 82% total variance observed, as study presented a wide variability in the protein expression per sample. We performed a PCA to identify protein expression components, suggesting for the first time that components two (prolidase) and three (XO and Nox2) of protein expression patterns in the synovia membrane may be involved in KOA (Figure 3).

In conclusion, we propose, with the aid of these data, that the increase in prooxidant proteins of synovial membrane may contribute to the inflammation state and therefore to the degradation of the articular cartilage. To the best of our knowledge, this study suggests that, besides XO and Nox2, the prolidase enzyme may be involved in ROS production in the synovial membrane and therefore in the oxidative stress involved in KOA disease. However, we are aware that further investigation is required to determine the mechanisms whereby ROS production arising from synovial membrane may regulate the chondrocyte metabolism in order to ascertain future therapeutic approaches for KOA treatment.

## Figures and Tables

**Table 1:** Demographic, anthropometric, and clinical characteristics.

Characteristic	All subjects	Controls	KOA patients	*p* value
Age (years)				
Median (interquartile range)	65 (19)	35 (17)	67 (16)	<0.01^*∗*^
BMI (Kg/cm^2^)				
Median (interquartile range)	27.34 (7.20)	25.65 (8.94)	27.47 (6.37)	0.48^*∗*^
Gender (%)				
Female	48 (71.64)	3 (30.00)	45 (78.95)	<0.01^*∗∗*^
Male	19 (28.36)	7 (70.00)	12 (21.05)	
White blood cell count (10^9^/L)				
Median (interquartile range)	6.41 (2.4)	6.26 (1.92)	6.41 (2.4)	0.78^*∗*^

Bold text denotes statistical significance.

^*∗*^Mann–Whitney *U* test *p* value.

^*∗∗*^Fisher's exact test *p* value.
