# Corrigendum to “The Overexpression of NALP3 Inflammasome in Knee Osteoarthritis Is Associated with Synovial Membrane Prolidase and NADPH Oxidase 2”

**DOI:** 10.1155/2017/7847602

**Published:** 2017-03-16

**Authors:** Denise Clavijo-Cornejo, Karina Martínez-Flores, Karina Silva-Luna, Gabriela Angélica Martínez-Nava, Javier Fernández-Torres, Yessica Zamudio-Cuevas, Mónica Guadalupe Santamaría-Olmedo, Julio Granados-Montiel, Carlos Pineda, Alberto López-Reyes

**Affiliations:** ^1^Synovioanalysis Molecular Laboratory, Instituto Nacional de Rehabilitación “Luis Guillermo Ibarra Ibarra”, Secretaria de Salud, Calzada Mexico-Xochimilco No. 289, Col. Arenal de Guadalupe, 14389 Tlalpan, Mexico City, Mexico; ^2^Musculoskeletal and Articular Ultrasound Diploma Course, Instituto Nacional de Rehabilitación “Luis Guillermo Ibarra Ibarra”, Secretaria de Salud, Calzada Mexico-Xochimilco No. 289, Col. Arenal de Guadalupe, 14389 Tlalpan, Mexico City, Mexico; ^3^Biological and Health Sciences PhD Program, Universidad Autónoma Metropolitana Iztapalapa, Avenida San Rafael Atlixco 186, Col. Vicentina, 09340 Iztapalapa, Mexico City, Mexico; ^4^Tissue Engineering, Cell Therapy and Regenerative Medicine Research Unit, Instituto Nacional de Rehabilitación “Luis Guillermo Ibarra Ibarra”, Secretaria de Salud, Calzada Mexico-Xochimilco No. 289, Col. Arenal de Guadalupe, 14389 Tlalpan, Mexico City, Mexico; ^5^Instituto Nacional de Rehabilitación “Luis Guillermo Ibarra Ibarra”, Secretaria de Salud, Calzada Mexico-Xochimilco 289, Col. Arenal de Guadalupe, 14389 Tlalpan, Mexico City, Mexico

In the article titled “The Overexpression of NALP3 Inflammasome in Knee Osteoarthritis Is Associated with Synovial Membrane Prolidase and NADPH Oxidase 2,” [1] an incorrect version of Figure  2(b) is published. The correct version is shown as follows.

## Figures and Tables

**Figure 2:**
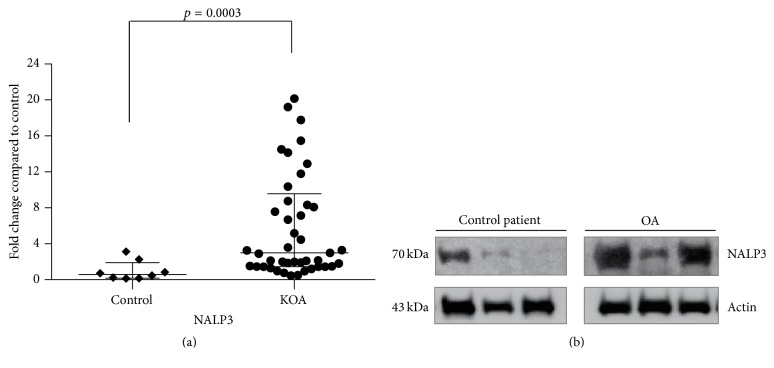
Inflammatory state present in KOA patients. Densitometry analysis of protein expression of NALP3 in comparison to the control group (a). Results are shown as the mean ± SEM (*p* < 0.001). Representative Western blot of protein levels relative to actin used as internal control (b).
